# System biology mediated assessment of molecular mechanism for sinapic acid against breast cancer: via network pharmacology and molecular dynamic simulation

**DOI:** 10.1038/s41598-023-47901-3

**Published:** 2023-12-11

**Authors:** Prarambh S. R. Dwivedi, C. S. Shastry

**Affiliations:** Department of Pharmacology, NGSM Institute of Pharmaceutical Sciences (NGSMIPS), Nitte (Deemed to be University), Mangalore, 575018 India

**Keywords:** Cancer, Computational biology and bioinformatics, Systems biology

## Abstract

Sinapic acid is a hydroxycinnamic acid widespread in the plant kingdom, known to be a potent anti-oxidant used for the treatment of cancer, infections, oxidative stress, and inflammation. However, the mode of action for its chemotherapeutic properties has yet not been unleashed. Hence, we aimed to identify potential targets to propose a possible molecular mechanism for sinapic acid against breast cancer. We utilized multiple system biology tools and databases like DisGeNET, DIGEP-Pred, Cytoscape, STRING, AutoDock 4.2, AutoDock vina, Schrodinger, and gromacs to predict a probable molecular mechanism for sinapic acid against breast cancer. Targets for the disease breast cancer, were identified via DisGeNET database which were further matched with proteins predicted to be modulated by sinapic acid. In addition, KEGG pathway analysis was used to identify pathways; a protein-pathway network was constructed via Cytoscape. Molecular docking was performed using three different algorithms followed by molecular dynamic simulations and MMPBSA analysis. Moreover, cluster analysis and gene ontology (GO) analysis were performed. A total of 6776 targets were identified for breast cancer; 95.38% of genes predicted to be modulated by sinapic acid were common with genes of breast cancer. The ‘*Pathways in cancer*’ was predicted to be modulated by most umber of proteins. Further, *PRKCA*, *CASP8*, and *CTNNB1* were predicted to be the top 3 hub genes. In addition, molecular docking studies revealed *CYP3A4*, *CYP1A1*, and *SIRT1* to be the lead proteins identified from AutoDock 4.2, AutoDock Vina, and Schrodinger suite Glide respectively. Molecular dynamic simulation and MMPBSA were performed for the complex of sinapic acid with above mentioned proteins which revealed a stable complex throughout simulation. The predictions revealed that the mechanism of sinapic acid in breast cancer may be due to regulation of multiple proteins like *CTNNB1*, *PRKCA*, *CASP8*, *SIRT1*, and cytochrome enzymes (*CYP1A1* & *CYP3A4*); the majorly regulated pathway was predicted to be ‘*Pathways in cancer*’. This indicates the rationale for sinapic acid to be used in the treatment of breast cancer. However, these are predictions and need to be validated and looked upon in-depth to confirm the exact mechanism of sinapic acid in the treatment of breast cancer; this is future scope as well as a drawback of the current study.

## Introduction

Cancer is one of the dreaded diseases portrayed by uncontrolled proliferation of abnormal cells that occurs due to impairment of cell division and apoptotic process^1^. Breast cancer (BC) is the most common cause of death among women, and it is divided into categories based on the organs involved. Blood leakage from the nipple, changes in the shape or texture of the nipple or breast, and a lump in the breast are indications for breast cancer^2^. Female breast cancer (11.7%) has surpassed lung cancer (11.4%) as the most commonly diagnosed cancer, with an estimated 2.3 million new cases^3^. The current scenario of breast cancer in the world briefs *approx.* 46 incidences of breast cancer for every 100,000 females with a mortality of around 13 women in 100,000^4^. Breast cancer etiology is majorly (> 90%) associated with environmental and lifestyle factors; the major risk factors include obesity, oral contraception, hormone replacement therapy (HRT), late menopause, early menarche, high calorie/fat diet, and alcohol consumption^5,6^.

The utilization of medicinal plants for the treatment of various diseases has a long history; contains multiple diverse metabolites which are pharmacologically active^7,8^. In the current scenario, the use of plants as a source of medicine has diminished; although prominent in the Asian subcontinent where traditional medicine systems like Traditional Chinese Medicine (TCM) and Ayurveda are still practiced^9,10^. A Widely used taxane-diterpene plant metabolite, Taxol (Paclitaxol) obtained from *Taxus brevifolia* bark is an integral bioactive for the current chemotherapy^11^. Similarly, Vincristine and Vinblastine are *Catharanthus roseus* isolates; used to treat clinical leukemia and Hodgkin’s disease^12^.

Sinapic acid is 1 of the 4 most common hydroxycinnamic acids which is widespread in the plant kingdom and is present in various fruits, vegetables, cereal grains, oil seed crops, some spices, and medicinal plants^13^. It has been proposed to be a potent anti-oxidant by researchers with its effectiveness to be greater than ferulic acid, a hydroxycinnamic acid already used as a natural antioxidant in foods, beverages, and cosmetics^14^. Balaji et al. reported sinapic acid to possess chemo-preventive potential against 1,2-dimethylhydrazine (DMH)-induced rat colon carcinogenesis^15^. Also, a study conducted by Eroğlu et al. reported the anti-cancer potential of sinapic acid against PC-3 and LNCaP human prostate cancer cell lines via overexpression of BAX, CASP3, CASP8, CYCS, FAS, TIMP-1, CDH1 and downregulation of MMP-9 in PC-3 cell lines^16^. In addition, multiple studies have been conducted to assess the cytotoxic potential of sinapic acid on various cell lines like human laryngeal carcinoma cell line (HEp-2)^17^, chinese hamster lung fibroblasts (V79), human cervical carcinoma (HeLa)^18^, and breast cancer cell lines MCF7, MDA-MB-231^19^, and T47D^20^ which displayed promising potential of sinapic acid as an anti-cancer agent. Hence, making sinapic acid a convincible bioactive against cancer; this additionally, kindled us to assess the potential targets sinapic acid modulates against breast cancer.

System biology tools own great importance in the current scenario of drug discovery as it provides precedence to identify a potent lead hit from multiple test agents^21,22^. Sinapic acid has been tested and reported against various pathological conditions such as infections, oxidative stress, inflammation, cancer, diabetes, neurodegeneration, and anxiety^23^. It has also been reported to possess protective properties against various chemotherapeutic drugs like doxorubicin, cisplatin, and methotrexate^24–26^. In this context, we have utilized system biology tools to predict the possible molecular mechanism for sinapic acid as an anti-cancer agent for the treatment of breast cancer via utilizing tools like molecular docking, gene set enrichment analysis, and gene ontology analysis.

## Material and methods

### Physiochemical properties and identification of targets

The physicochemical properties of sinapic acid were retrieved from the Molsoft LLC database (https://molsoft.com/mprop/) and parameters like number of hydrogen bond donors (NHBD) & acceptors (NHBA), octanol/water partition coefficient (MolLogP), and drug-likeness score were predicted. The reported targets of breast cancer were retrieved from DisGeNET database (https://www.disgenet.org/) using the keyword “*Breast Carcinoma*” with disease id “*C0678222*”; 6776 genes were identified. The targets regulated by test agent “Sinapic acid” were retrieved from DIGEP-Pred^27^ (http://www.way2drug.com/ge/) database via previously retrieved SMILES from PubChem (https://pubchem.ncbi.nlm.nih.gov/compound/637775); targets possessing pharmacological activity greater than pharmacological inactivity were included.

### Gene ontology (GO) analysis

The gene ontology (GO) of targets modulated by sinapic acid were retrieved from the STRING database; cellular components (CC), molecular function (MF), and biological process (BP) were retrieved for individual analysis. A chord diagram was constructed for the top 5 CC, MF, and BP using the software OriginPro 2021b. Further, GO analysis was performed where a correlation matrix was implemented with two-tailed test for confidence of 95% to compute the Pearson correlation coefficient.

### Network construction

The bioactive regulated targets identified from DIGEP-Pred were matched with the genes involved in the pathogenesis of breast cancer (*DisGeNET database code: C0678222*) and a network of protein–protein interaction was constructed via STRING^28^ *ver. 11.5* (https://string-db.org/). Further, KEGG pathway analysis was utilized to identify sinapic acid-regulated pathways which were further integrated into pathway-protein interaction via Cytoscape^29^ *ver*. *3.9.0*. The network was treated as directed and analyzed based on edge count by mapping node size and color. Additionally, network analysis was done based on “*Node degree distribution*” and “*Betweenness by degree*” where parameters like eccentricity, neighborhood connectivity, in-degree distribution, and outdegree distribution were analyzed.

### Construction of cluster and its analysis

Cluster analysis was performed via ClueGo^30^ add-on tool (*v.2.5.8*) in Cytoscape *3.9.0*; CC, MF, BP, and KEGG enriched proteins were analyzed by applying functional analysis (two-sided hypergeometric test) with ‘Network specificity’ to be ‘medium’, pV value to be less than 0.05, the ‘GO tree interval’ was kept in the range of ‘3–8 pathway’, and ‘GO term selection of cluster’ was set to three genes minimum with a percentage of 4. Kappa score threshold was kept at 0.4 using the Bonferroni step-down correction method. The cluster was constructed on ClueGo layout with the above parameters.

### In-silico molecular docking

In the present study, sinapic acid was docked with 62 proteins using 3 different algorithms; AutoDock Vina, AutoDock 4.2, and Schrodinger suite Glide. Initially, the ligand was prepared by minimizing its energy, followed by target preparation and molecular docking. In addition, docking was performed on the top 5 targets with their respective standard ligands using the three algorithms.

#### Ligand preparation

The 3D conformation of sinapic acid was retrieved in .sdf format from the PubChem database and was converted into .pdb format using Discovery studio visualizer (BO*VIA* Discovery Studio Visualizer; https://discover.3ds.com/discovery-studio-visualizer-download) 2019. The energy of the ligand was minimized using MMFF97 forcefield^31^ and was converted into .pdbqt format for docking.

#### Homology modeling and target preparation

The structures of targets were initially queried in UniProt (https://www.uniprot.org/) database to identify available structures in Protein Data Bank (RCSB; https://www.rcsb.org/). The targets not available were further modeled using the known FASTA sequence deposited in UniProt database using SWISS-MODEL^32^ (https://swissmodel.expasy.org) (Supp. File 4 (Sheet 1)). All the hetero-atoms present in the protein were removed and saved in .pdb format utilizing Discovery studio visualizer.

#### Ligand–protein docking

##### AutoDock vina

The ligand sinapic acid was docked against the identified proteins using AutoDock Vina at PyRx (https://pyrx.sourceforge.io/) *ver. 0.8* platform to obtain binding affinity of sinapic acid with multiple targets involved in the pathogenesis of breast cancer. Further, the targets with which sinapic acid possessed the least binding energy were visualized in Discovery Studio Visualizer and the poses of ligand pertaining to the least binding energy with maximum intermolecular interactions was selected to be foreseen in Discovery studio.

##### AutoDock 4.2

The proteins were read in .pdb format, previously created via Discovery studio by removing hetero atoms and unwanted chains (if any). Polar hydrogens were added along with Kollman charges to build the protein into .pdbqt. The grid box was set at the center of the macromolecule and docking was performed using a genetic algorithm with 10 genetic algorithm runs with two-point crossover mode. The pose possessing the least binding energy was visualized for protein–ligand interaction.

### Schrodinger suite glide

The LigPrep module of Schrodinger suite (https://www.schrodinger.com/) 2019 was used to prepare the ligand molecule, a low-energy conformation of the ligand was made prior to docking. Further, the protein preparation wizard module was utilized to prepare the protein by adding missing amino acids and the OPLS-3 forcefield was used to minimize the energy. Water molecules were removed and conceivable ionization states for the hetero atoms in the protein were produced; one with the highest stability was chosen.

The site map module was used to identify the binding pocket of the proteins, and a grid box was set with dimensions (18 Å, 18 Å, 18 Å) using the Glide grid generation wizard (https://nodepit.com/node/com.schrodinger.knime.node.gridgen.GridGenNodeFactory) at the center of the identified binding pocket. The Glide module of Schrodinger suite 2019 was used to perform protein–ligand docking using extra precision (XP) mode and OPLS-3 force field was implemented to dock in flexible docking mode; creating confirmations for the input ligand. Further, the Docking score was generated automatically after the completion and results were analyzed via the Glide modules XP visualizer.

### Molecular dynamic simulation

#### Reason for selection of proteins

The complexes subjected to MD simulation were chosen based on the hub genes (based on edge count) identified from gene enrichment analysis (*PRKCA* (PDB ID: 3PFQ), *CASP8* (PDB ID: 1QTN), and *CTNNB1* (PDB ID: 2Z6H)) and the complexes possessing the least binding energy with respective algorithms *i.e.* AutoDock 4.2 (*CYP3A4* (PDB ID: 5A1R)), AutoDock Vina (*CYP1A1* (PDB ID: 4I8V)), and Schrodinger (*SIRT1* (PDB ID: 4ZZI)).

Gromacs version 2022.1 (https://www.gromacs.org/) was used to perform the MD Simulations. The topology of the protein was generated by applying charmm36 forcefield^33^ using the pdb2gmx module of gromacs. The proteins were solvated using three-point water model in a dodecahedron box with dimensions of 1 nm in all directions. The model system was neutralized by adding sodium (Na^+^) and Chloride (Cl^-^) ions as counter ions. Energy minimization was performed using steepest descent integrator with verlet cutoff scheme for a maximum of 50,000 steps to achieve the least energy confirmation. The system was equilibrated using Canonical (NVT) and Isobaric (NPT) for 100 picoseconds. V-rescale, a modified Berendsen thermostat was applied to maintain constant volume and temperature at 300 K. Similarly, a C-rescale pressure coupling algorithm was applied to maintain constant pressure at 1 bar. Particle Mesh Ewald was applied for computing long-range electrostatics, coulomb, and vander waals with a cut-off of 1.2 nm. The LINCS algorithm was used to constrain bond length. Each complex was subjected to MD run for 200 ns; the coordinates and energies were saved at every 20 picoseconds. The trajectories generated were analyzed using in-built gromacs utilities^34^.

### Molecular mechanics Poisson-Boltzmann surface area (MMPBSA) analysis

The gmx_MMPBSA module^35^ was used to analyze the energy contribution parameters like Vander Waals & electrostatic molecular mechanics energy, polar contribution to the salvation energy, non-polar contribution of solute–solvent interactions to the solvation energy**,** non-polar contribution of attractive solute–solvent interactions to the salvation energy, total gas phase molecular mechanics energy, total solvation energy, and total relative binding energy**.**

The MMPBSA run was performed for 82 frames from a total of ten thousand frames with an interval of 120. The Poisson Boltzmann calculations were performed using an internal PBSA solver in a sander. The MMPBSA_ana module was used to visualize the results obtained from the gmx_MMPBSA run^36^.

## Results

### Physiochemical properties and Identification of targets

**Figure 1:**
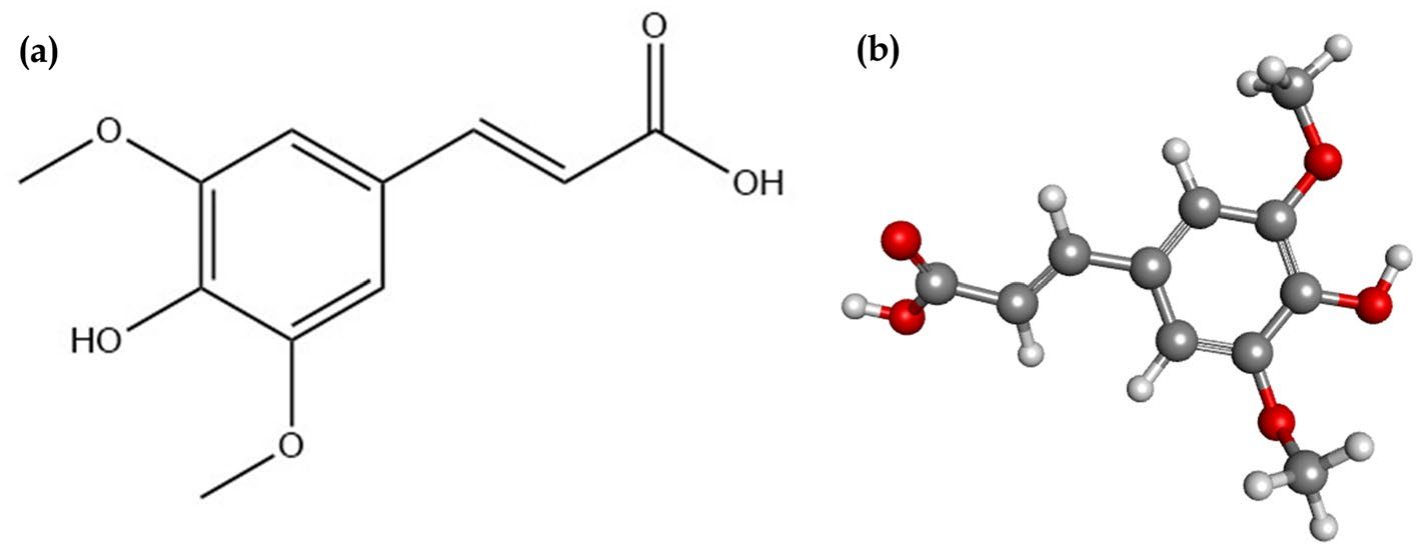
(**a**) 2D and (**b**) 3D structure of sinapic acid; PubChem CID: 637,775, Molecular formula: C_11_H_12_O_5_, Molecular weight: 224.21 g/mol, NHBD: 2, NHBA: 5, MolLogP: 1.09, and drug-likeness score: – 1.07.

**Figure 2:**
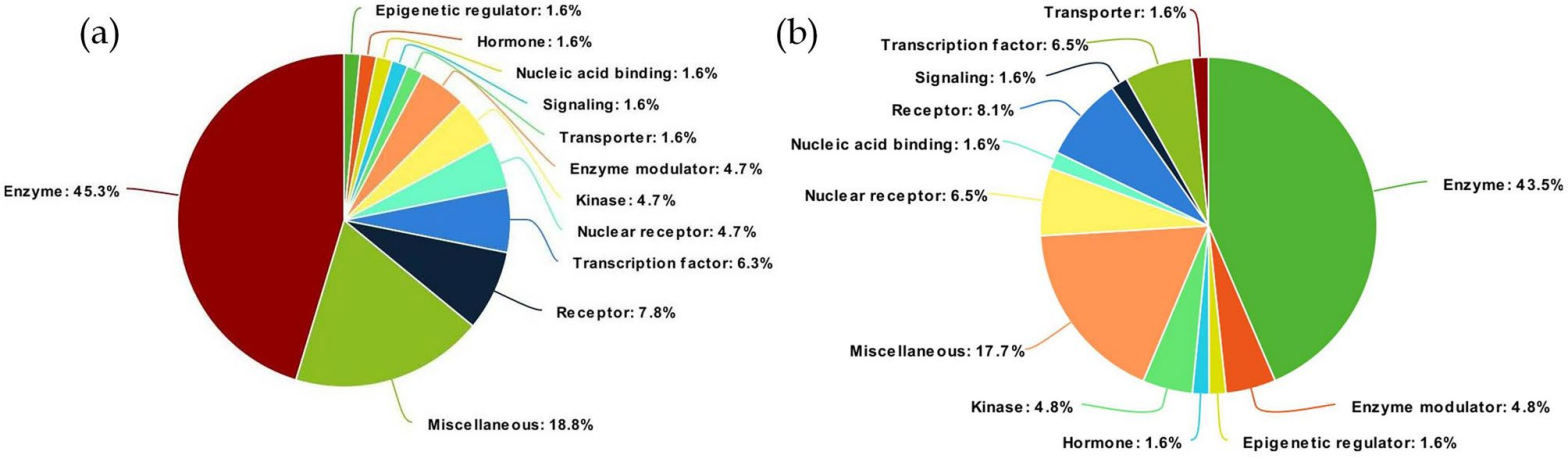
(**a**) Classification of targets identified in the pathogenesis of breast cancer, (**b**) Classification of targets modulated by sinapic acid which are involved in the pathogenesis of breast cancer.

The physiochemical properties of sinapic acid revealed NHBD as 2, NHBA as 5, MolLogP as 1.09, and drug-likeness score of − 1.07; sinapic acid has a molecular weight of 224 g/mol and obeys Lipinski’s rule of five (Fig. 1). 6776 targets related to breast cancer were identified from which 45.3% of the overall targets belonged to the class of enzymes, 7.8% were identified as receptors, and 6.3% of targets were identified as a transcription factors (Fig. 2). Similarly, the proteins predicted to be modulated by sinapic acid were majorly classified as enzymes (43.5%), and receptors (8.1%) (Fig. 2).

### Gene enrichment and network analysis

**Figure 3:**
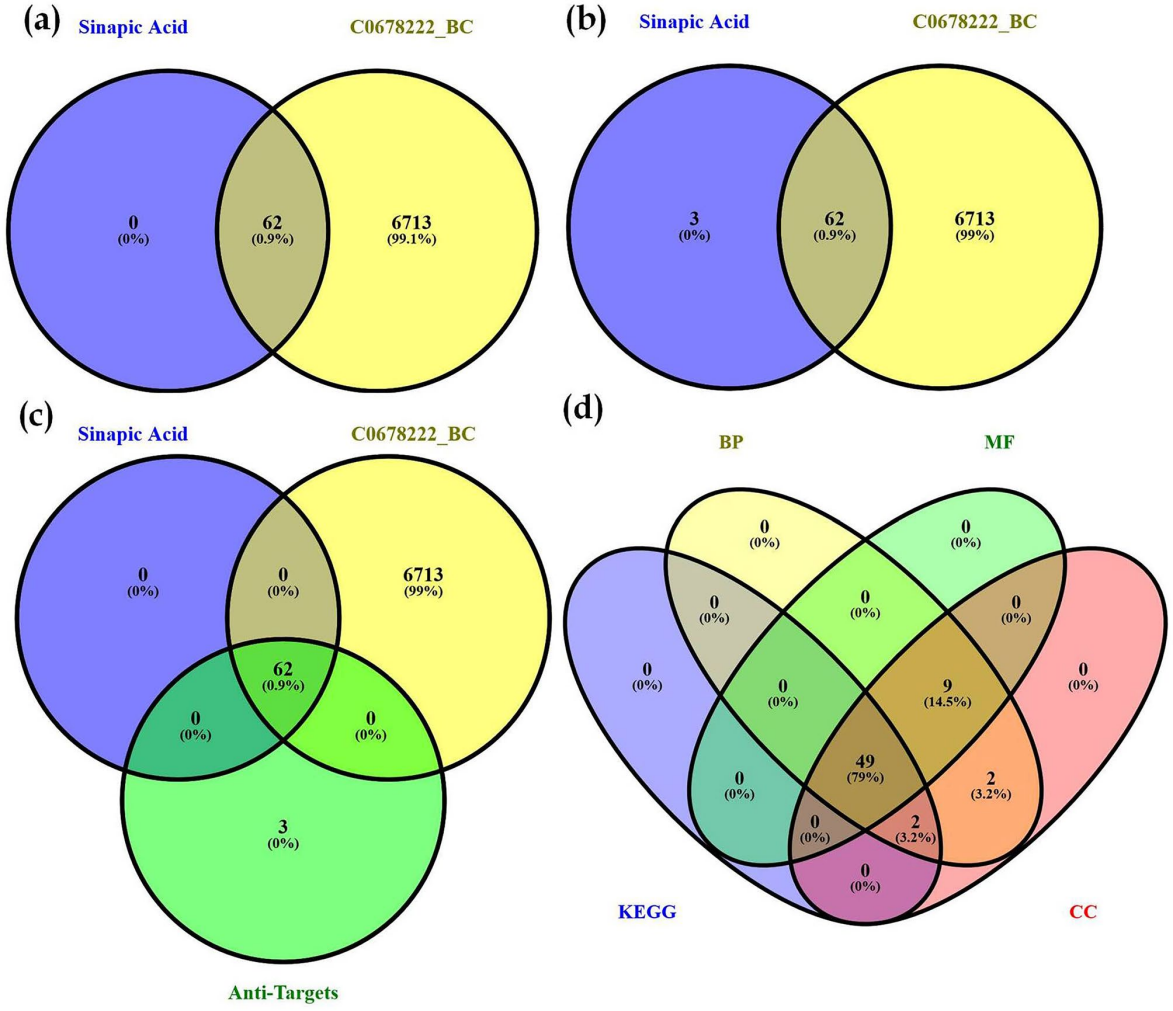
Venn diagram representation of (**a**) targets involved in breast cancer (C0678222) *vs* matched targets, (**b**) targets involved in breast cancer (C0678222) *vs* targets regulated by sinapic acid, (**c**) targets of sinapic acid vs targets involved in breast cancer (C0678222) vs anti-targets, (**d**) GO terms (cellular component, molecular function, and biological process) *vs* KEGG mediated genes.

**Figure 4:**
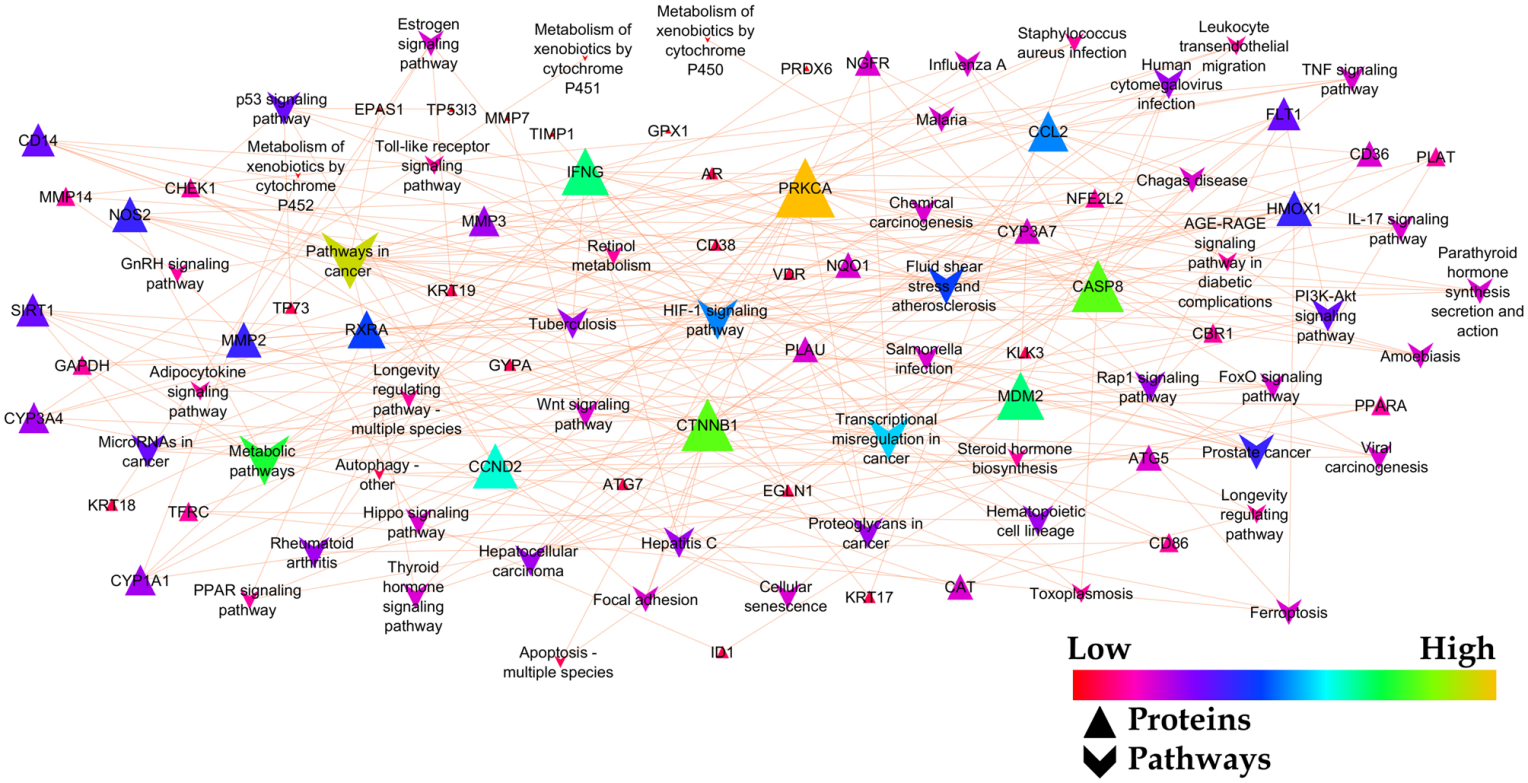
Protein-pathway interaction of genes modulated by sinapic acid and pathways identified to be involved.

The prediction of targets modulated by sinapic acid were retrieved from the DIGEP-Pred database where we identified 65 proteins possessing Pa > Pi from which 62 proteins were found to match with proteins identified for the pathogenesis of breast cancer (Fig. 3). The gene enrichment analysis predicted 95.38% of genes modulated by sinapic acid to be involved in breast cancer and the remaining 4.6% were predicted to be anti-targets (*SELL*, *GCLM*, and *GSS*) (Fig. 3). STRING was used to assess protein–protein interaction which was further integrated with KEGG pathway analysis where 50 pathways were identified (Supp. file 1 (sheet 1); Table 1). A network between pathways and proteins was constructed using Cytoscape based on edge count; “*Pathways in cancer*” was predicted to be the majorly modulated pathway. Further, *PRKCA*, *CASP8*, and *CTNNB1* were predicted to be the top 3 hub genes to be regulated by sinapic acid (Fig. 4). Additionally, “*cellular senescence*” was identified to possess the maximum “*edge betweenness*” of 102 followed by “*chemical carcinogenesis*” with an *edge betweenness* of 101 (Supp. file 1 (sheet 2)). “*Pathways in cancer”* and *PRKCA* displayed the highest *indegree* and *outdegree* distribution of 17 and 16 respectively. Similarly, “*Human cytomegalovirus infection*” was predicted to possess the highest neighborhood connectivity of 13.2 (Supp. file 1 (sheet 3)).

**Table 1:**
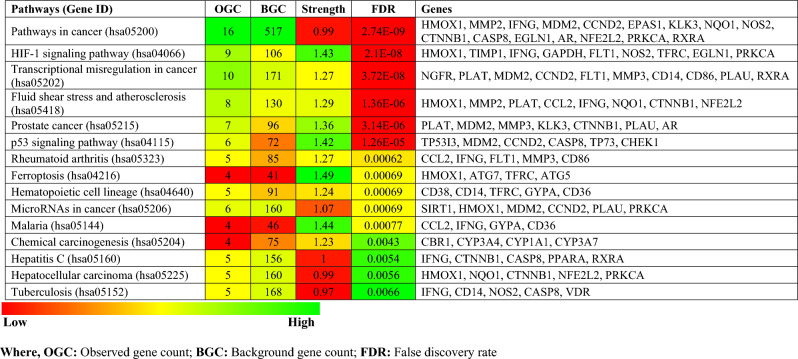
Top 15 KEGG pathways modulated by sinapic acid against breast cancer.

*OGC* observed gene count, *BGC* background gene count, *FDR* false discovery rate.

### Gene ontology

**Figure 5:**
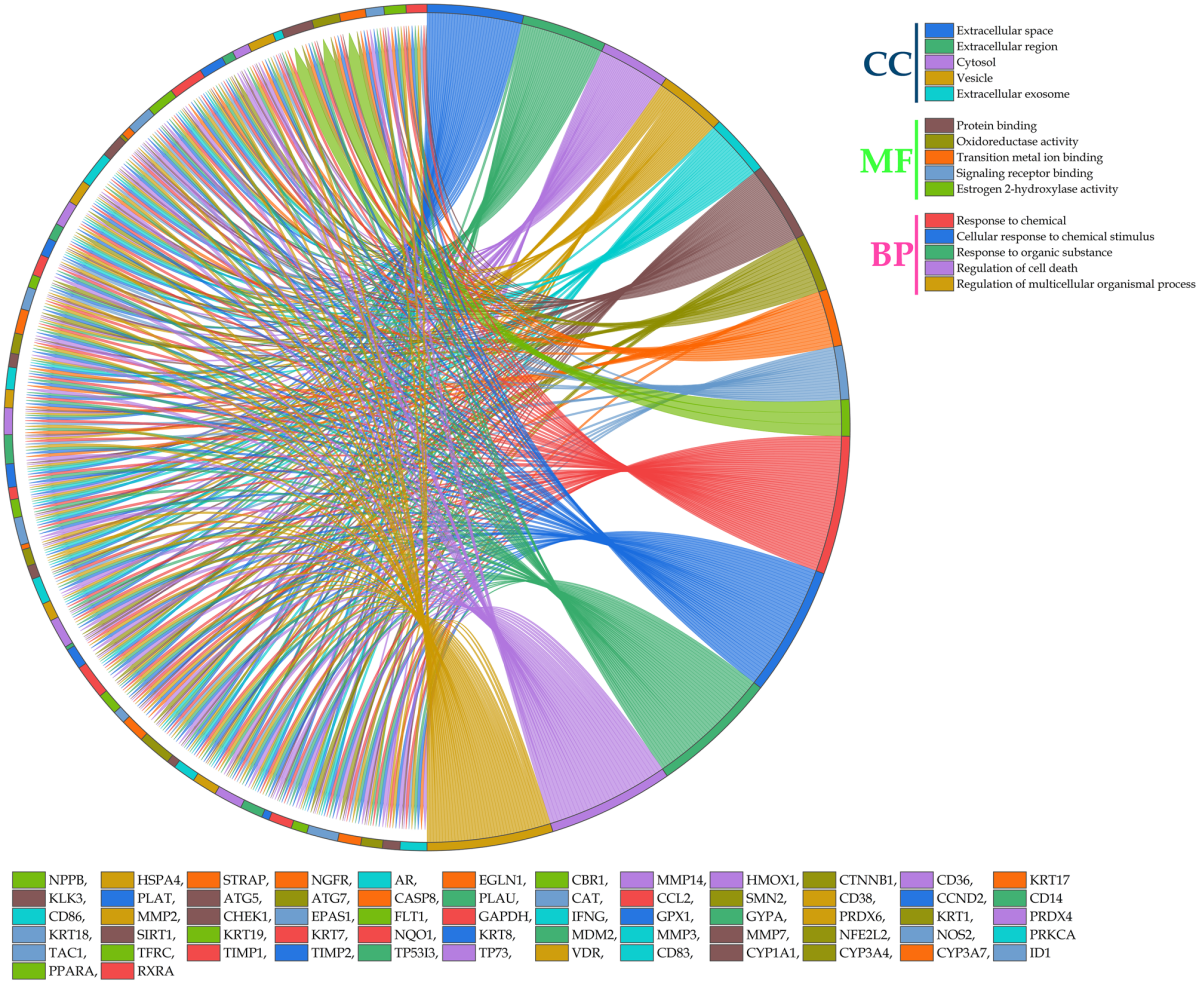
Chord diagram representation of top 5 GO terms belonging to cellular components (CC), molecular function (MF), and biological process (BP).

The data for GO terms i.e. cellular components (CC), molecular function (MF), and biological process (BP) were retrieved from the STRING database. GO analysis identified 23 CC in which extracellular space (GO:*0005615*) scored the lowest false discovery rate of 1.34E-08 via the modulation of 34 observed genes i.e.* HMOX1*, *TIMP1*, *MMP2*, *PLAT*, *CCL2*, *CD38*, *IFNG, GAPDH, CAT, KRT1, MMP7, TIMP2, FLT1, CBR1, MMP3, HSPA4, CD14, MMP14, KLK3, TAC1, KRT7, CD86, PRDX6, CTNNB1, TFRC, KRT19, PLAU, NPPB, PRDX4, KRT18, CHEK1, CD36, PRKCA,* and *KRT8* against 3195 background genes at a strength of 0.53. Similarly, 32 MF were identified in which protein binding (GO:*0005515*) scored the lowest false discovery rate of 5.26E-06 via the modulation of 46 observed genes i.e.* NGFR, SIRT1, HMOX1, TIMP1, PLAT, CCL2, CD38, IFNG, GAPDH, TP53I3, CAT, KRT1, MDM2, CCND2, TIMP2, EPAS1, FLT1, MMP14, NQO1, TAC1, NOS2, CYP3A4, PRDX6, CTNNB1, ATG7, CASP8, TFRC, GYPA, EGLN1, AR, ID1, NPPB, TP73, CYP1A1, SMN2, KRT18, NFE2L2, PPARA, CHEK1, STRAP, CD36, GPX1, PRKCA, RXRA, VDR,* and *KRT8* against 7026 background genes at a strength of 0.32. Moreover, 550 BP were identified where, response to chemical (GO:*0042221*) scored the lowest false discovery rate of 7.45E-16 via the modulation of 48 observed genes i.e.* NGFR, SIRT1, HMOX1, TIMP1, MMP2, CCL2, CD38, IFNG, GAPDH, CAT, MDM2, TIMP2, EPAS1, FLT1, MMP3, HSPA4, CD14, MMP14, NQO1, TAC1, NOS2, CD86, CYP3A4, PRDX6, CTNNB1, ATG7, CASP8, TFRC, KRT19, EGLN1, ATG5, PLAU, AR, ID1, TP73, CD83, PRDX4, CYP1A1, KRT18, NFE2L2, PPARA, CD36, GPX1, PRKCA, RXRA, VDR, KRT8,* and *CYP3A7* against 4333 background gene count with a strength of 0.54 (Supp. file 2). The GO of the top 5 CC, MF, and BP has been represented in the form of a chord diagram (Fig. 5). The integration of GO terms with KEGG modulated proteins predicted 79% of the genes to be involved in both the GO terms as well as KEGG modulated proteins (Fig. 3).

**Figure 6:**
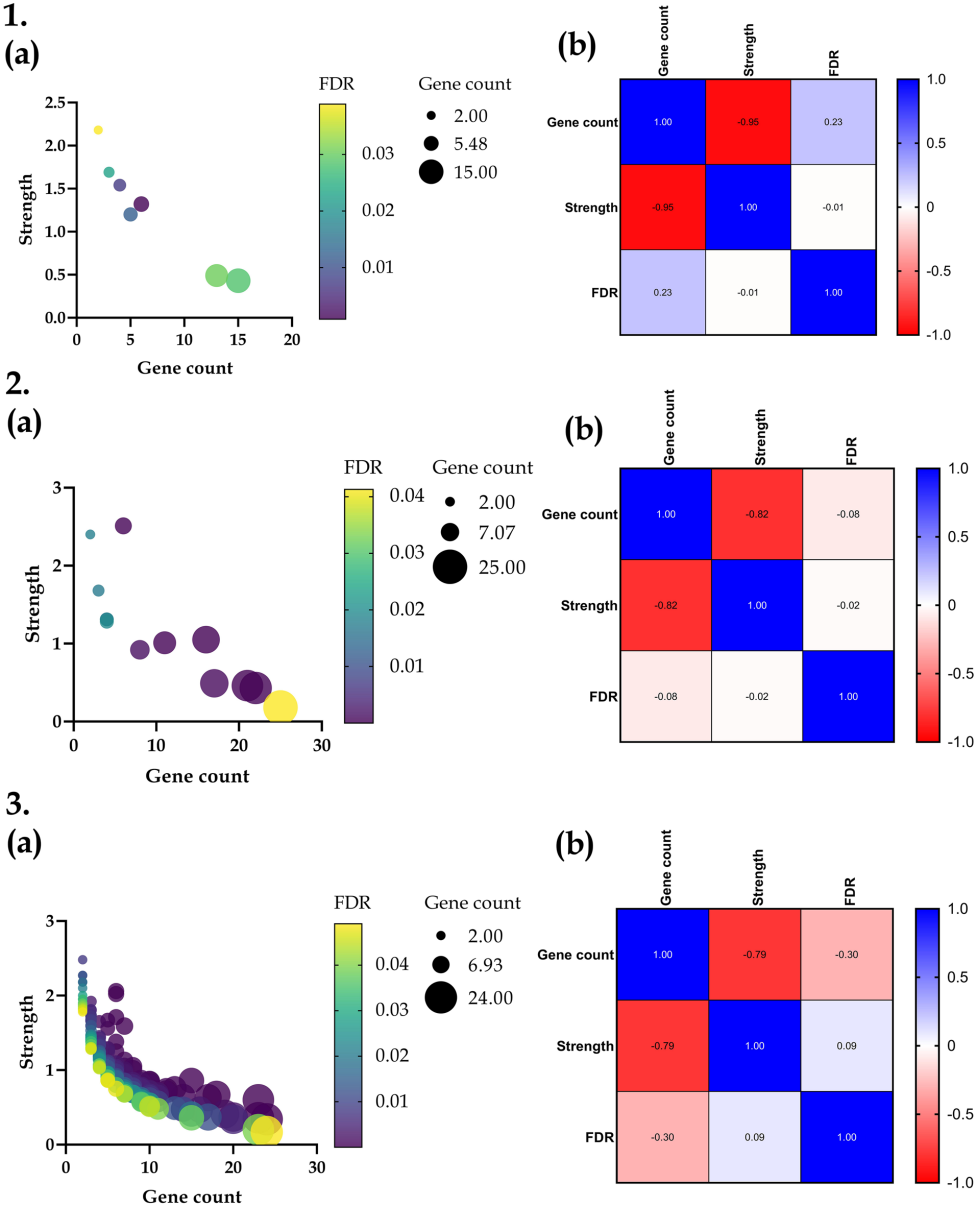
Correlation matrix analysis of GO terms (**1**) Cellular components, (**2**) Molecular function, (**3**) Biological process where (**a**) represents the correlation between Strength *vs* Gene count *vs* False discovery rate as bubble colour map, (**b**) represents the Pearson’s r coefficient of Gene count vs Strength vs False discovery rate as a heat map.

Additionally, GO analysis by Pearson correlation matrix for CC; predicted the confidence interval of Pearson’s r coefficient for Gene Count (GC) vs Strength & false discovery rate (FDR) to be − 0.9932 to − 0.7050 & − 0.6301 to 0.8392 with p values 0.0009 & 0.614 respectively, Strength vs GC & FDR to be − 0.9932 to − 0.7050 & − 0.7589 to 0.7471 with p values 0.0009 & 0.977 respectively, and FDR vs GC & strength to be − 0.6301 to 0.8392 & − 0.7589 to 0.7471 with p values 0.6137 & 0.9768 respectively with a sample size of 7. Similarly, for MF the confidence interval of r for GC vs Strength & FDR was predicted to be − 0.9427 to − 0.4793 & − 0.6063 to 0.4903 with p values 0.0007 & 0.787 respectively, Strength *vs* GC & FDR to be − 0.9427 to − 0.4793 & − 0.5649 to 0.5367 with p values 0.0007 & 0.948 respectively, and FDR vs GC & strength to be − 0.6063 to 0.4903 & − 0.5649 to 0.5367 with p values 0.7871 & 0.9476 respectively with a sample size of 13. Moreover, for BP the confidence interval of r for GC vs Strength & FDR was predicted to be − 0.8401 to − 0.7228 & − 0.4338 to − 0.1554 with p values 3.08719507014992e-036 & 0.00009 respectively, Strength *vs* GC & FDR to be − 0.8401 to − 0.7228 & − 0.06039 to 0.2426 with p values 3.08719507014992e-036 & 0.2334 respectively, and FDR *vs* GC & strength to be − 0.4338 to − 0.1554 & − 0.06039 to 0.2426 with p values 0.00008 & 0.2334 respectively with a sample size of 165 (Fig. 6).

### Cluster analysis

Cluster analysis was performed via the ClueGo addon tool in Cytoscape *ver. 3.9.0* with CC, MF, BP, and KEGG enriched genes. The analysis displayed 1 cluster with 38 groups and 60 (96.77%) identified genes were involved in the cluster (Supp. file 3 (sheet 1)); 201 GO terms were identified with 522 connections. Additionally, 115 GO edges possessed a kappa score of 2, 163 edges possessed a kappa value of 1, and 68 edges with a kappa score above 0.8 indicating majority of the data to be reliable and accurate (Supp. file 3 (sheet 2)). Cellular response to oxidative stress (group 37) possessed the highest percent of genes/group with 36 genes ((Supp. file 3 (sheet 3)). The kappa score matrix generated by ClueGO has been depicted in (Supp. file 3 (sheet 4)). The Supp. file 5 represents cluster embraced with GO terms and KEGG enriched proteins.

### Molecular docking

**Figure 7:**
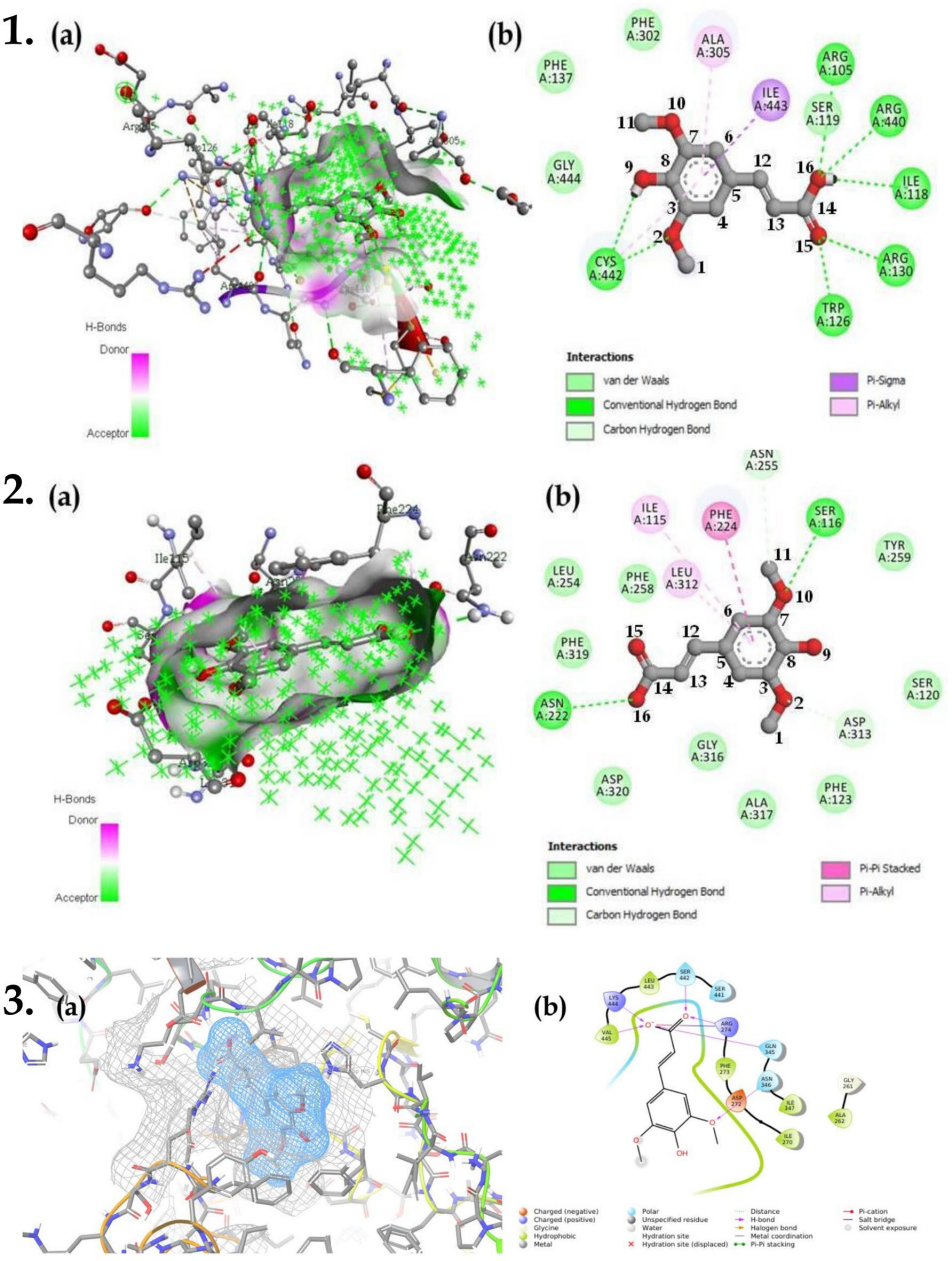
(**a**) 3D and (**b**) 2D interaction of (**1**) sinapic acid with *CYP1A1* when docked in AutoDock 4.2; (2) sinapic acid with *CYP1A1* when docked in AutoDock Vina; (3) sinapic acid with *SIRT1* when docked in Schrodinger suite Glide.

Molecular docking via AutoDock 4.2 revealed proteins *CYP3A4*, *CYP3A7*, and *SIRT1* to possess the least binding energy of − 7.6, − 7.5, and − 6.8 kcal/mol with 7 hydrogen bonds for each cytochrome enzymes and 4 hydrogen bonds for *SIRT1*. *CYP3A4* possessed 7 hydrogen bonds with amino acids *CYS442*, *ARG105*, *ARG440*, *ILE118*, *ARG130*, and *TRP126*; *CYS422* formed a hydrogen bond with the oxygen at position 2 and hydroxyl of position 9, *ARG105*, *ARG440*, and *ILE118* formed hydrogen bonds with hydroxyl group placed at 16^th^ position. Similarly, *TRP126* and *ARG130* formed hydrogen bonds with the oxygen placed at position 15. In addition, there were 3 π-bond interactions and 4 Vander Waal interactions with amino acids *ALA305*, *ILE443*, *CYS442,* and *PHE302*, *PHE137*, *GLY444*, *SER119* respectively (Fig. 7; Supp. file 4 (sheet 2)).

Molecular docking via AutoDock vina revealed *CYP1A1*, *CAT*, and *NOS2* to possess the least binding energy of − 7.6, − 7.4, and − 7.1 kcal/mol with 2 and 4 hydrogen bonds respectively for *CYP1A1* and *CAT*. *NOS2* possessed zero hydrogen bonds with 10 π-bond interactions and 5 Vander Waal interactions. *CYP1A1* formed 2 hydrogen bonds with *SER116* and *ASN222*, where *SER116* formed hydrogen bond with the oxygen at the 2nd position and *ASN222* formed a hydrogen bond with oxygen in hydroxyl group at the 16th position. Additionally, *CYP1A1* formed 9 π-bond residues with residues *ASP313*, *ALA317*, *PHE224*, *PHE123*, *ASN225*, *LEU312*, and *ILE115* and 7 Vander-Waal interactions with *SER120*, *GLY316*, *TYR259*, *PHE258*, *ASP320*, *PHE319*, and *LEU254* (Fig. 7). Similarly, *CAT* formed 4 hydrogen bonds with *HIS75* and *SER114* with oxygen at position 15, *GLY147* with hydroxyl group at position 16, and *HIS362* at position 2 (Supp. file 4 (sheet 3)).

Docking in glide displayed *SIRT1*, *NOS2*, and *VDR* to possess the least docking score of -6.86, -6.57, and -6.51 kcal/mol with 6, 1, and 1 hydrogen bond respectively. *SIRT1* formed hydrogen bonds with amino acids *LYS444*, *GLN345*, and *VAL445* with hydroxyl at position 16, *SER442* and *ARG274* formed hydrogen bond with oxygen at position 15, and *ASN346* formed a bond with oxygen at position 10. In addition, *SIRT1* possessed one salt bridge between *ARG274* and hydroxyl at position 16 (Fig. 7). Similarly, *NOS2* possessed 1 hydrogen bond with *ASN370* and hydroxyl present at position 8. Moreover, 3 π-π stacking were visualized with the benzene ring and 2 amino acids (*TRP194* and *PHE369)*. Likewise, *VDR* possessed 1 hydrogen bond between *SER237* and hydroxyl group at position 9 (Supp. file 4 (sheet 4)).

### Molecular stability of docked complexes

#### Sinapic acid-protein kinase C-α complex

**Figure 8:**
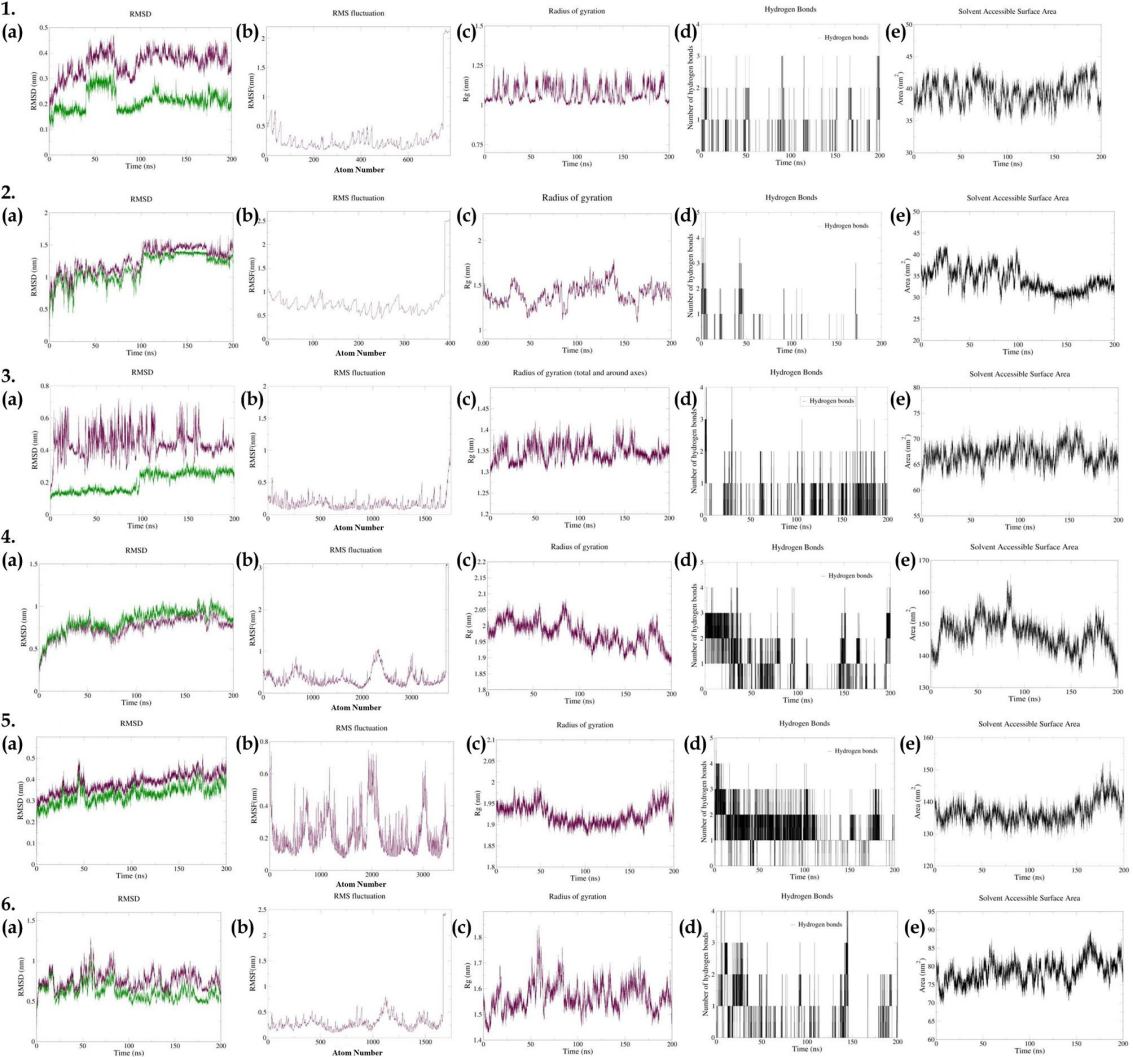
Parameters describing stability of sinapic acid complex with proteins (**1**) *PRKCA*; (**2**) *CASP8*; (**3**) *CTNNB1*; (**4**) *CYP3A4*; (**5**) *CYP1A1*; (**6**) *SIRT1*. Where, (**a**) RMSD of backbone (purple) and complex (green); (**b**) RMSF; (**c**); Radius of gyration; (**d**) Number of hydrogen bonds between protein and ligand; and (**e**) Solvent Assessable Surface Area (SASA).

On completion of MD run the RMSD value was analyzed for the backbone as well as complex which was not stable at the beginning of the MD run up to 45 ns and possessed fluctuation between 1 Å to 2.8 Å. The RMSD value remained unstable till 75 ns and displayed a high peak in RMSD at 70 ns. Further, there was a drop in the RMSD after 75 ns which gradually increased up to 120 ns and became stable at 130 ns with a value of ~ 2 Å for complex and ~ 4 Å for backbone; there was a difference of ~ 2 Å observed between RMSD of the backbone and complex. The RMSF value did not display major fluctuations and was in the range of ~ 1 Å to ~ 6 Å. The radius of gyration (Rg) displayed uniformity throughout the simulation with minor fluctuations in the range of ~ 1 Å to ~ 3 Å. The number of hydrogen bonds being formed throughout the simulation was analyzed; a maximum of 4 hydrogen bonds were formed. However, there was no constant bond formation between the ligand and protein. The solvent-accessible surface area helps to identify a free surface for the ligand to bind with protein; was found to be in the range of 35 to 45 nm^3^ throughout the simulation (Fig. 8).

#### Sinapic acid-caspase 8 complex

The RMSD of protein–ligand complex was found to be stabilized at ~ 105 ns where a spike increase in the RMSD was observed for backbone and complex. The RMSD fluctuation between 105 to 135 ns was ~ 3 Å thereafter it displayed a stable RMSD possessing fluctuations in a range of ~ 2 Å with a slight drop at 175 ns and constant thereafter; there was a difference of ~ 2 Å observed between the RMSD of backbone and complex. The RMSF values ranged between ~ 5 Å to ~ 10 Å throughout the simulation. The radius of gyration (Rg) value ranged from ~ 11 Å to ~ 16 Å throughout the simulation and became stable after 170 ns for the rest of the MD run. The highest number of hydrogen bonds formed between the ligand and protein was 5. However, there were no constant hydrogen bonds seen throughout the MD run. Moreover, the solvent-accessible surface area displayed a varied range of values till 100 ns simulation run; thereafter, a slight decrease in the SASA value from 40 nm^2^ to 35 nm^2^ was observed (Fig. 8).

#### Sinapic acid-β-catenin 1 complex

The RMSD of β-catenin 1-sinapic acid complex ranged from ~ 1 Å to ~ 6 Å throughout the MD run; the RMSD became stable for the backbone and complex after 100 ns of MD run thereafter the difference between the RMSD of backbone and complex was ~ 1 Å to ~ 2.5 Å. The RMSF value of the complex ranged from ~ 1 Å to ~ 5 Å, there were slight spikes visible at atoms ~ 1450 which may be due to hydrogen bonds being formed by the amino acids with sinapic acid. The radius of gyration stabilized after 50 ns and was in the range of 13 Å to 14 Å; the Rg value was observed to possess a deviation of 0.5 Å after 150 ns of MD run indicating stable protein–ligand interaction. A maximum of 4 hydrogen bonds were formed by the protein and ligand. The number of continuous hydrogen bonds increased after 100 ns and an increase in number of hydrogen bonds was observed till 200 ns of MD run. The solvent-accessible surface area was found to be in the range of 62 nm^2^ to 70 nm^2^ and there were observed fluctuations in SASA ranging from 130 to 200 ns (Fig. 8).

#### Sinapic cid-cytochrome 3A4 complex

The RMSD of *CYP3A4*-sinapic acid complex became stable after 50 ns, after which the RMSD value ranged from ~ 7 Å to ~ 10 Å throughout the simulation. There was a fluctuation in RMSD at ~ 175 ns; the difference between the RMSD of backbone and complex was observed to be ~ 1.5 Å. The RMSF value ranged between ~ 2 Å to ~ 10 Å throughout the simulation, there was increased spikes visible at atoms ~ 2100 to ~ 2400 which may be involved in the formation of bonds with the ligand. Additionally, the radius of gyration displayed fluctuation between the range ~ 19.0 Å to ~ 20.5 Å and was stable throughout the run. Moreover, a highest of 5 hydrogen bonds were formed between the protein and ligand; 3 constant hydrogen bonds were formed till 30 ns of MD run, thereafter the number of hydrogen bonds decreased to 1–2 bonds visible up to 80 ns. In addition, there was a fluctuation in the number of hydrogen bonds formed till 150 ns and no interaction was visible. Thereafter 150 ns of MD run 2–3 hydrogen bonds were visible which became constant with 3 hydrogen bonds at 190 ns. Initially, there was a decrease in the solvent-accessible surface area till 30 ns of simulation which further increased from 140 nm^2^ to 150 nm^2^. Later, the SASA value decreased as a stable complex was formed; SASA values varied throughout the simulation depending upon the stability of the complex. The SASA value ranged between 135 to 165 nm^2^ throughout the simulation (Fig. 8).

#### Sinapic acid-cytochrome 1A1 complex

The RMSD of protein ligand complex remained stable throughout the simulation after 50 ns of stability time of the complex. The RMSD value ranged from ~ 2 Å to ~ 4.5 Å throughout the simulation with a difference of ~ 0.5 Å was observed between backbone and complex. The RMSF value ranged from ~ 1 Å to ~ 7 Å throughout the simulation; high peaks of ~ 5Å to ~ 7Å were observed at atoms ~ 1250, ~ 2000, and ~ 3000 indicating that these residues may be responsible for the hydrogen bonds formed. The radius of gyration value ranged from ~ 18.7 Å to ~ 20 Å throughout the MD run; there was a slight decrease in the Rg value after 50 ns of stabilization period of the complex thereafter the Rg value was in the range of ~ 18.8 Å to ~ 19.4 Å indicating a difference of 0.6 Å. The Rg value remained unstable after 150 ns which may be due to the breakage of bonds formed between protein and ligand. A highest of 5 hydrogen bonds were formed 2 hydrogen bonds remained constant throughout the MD run till 110 ns thereafter there only 1 hydrogen bond was constant throughout the run. The solvent-accessible surface area remained constant throughout the MD run with a range of 73 Å to 85Å. There was an increase in the SASA value after 150 ns (Fig. 8).

#### Sinapic acid-sirtuin 1 complex

The RMSD of sirtuin 1-sinapic acid complex was assessed to be in the range of ~ 5 Å to ~ 10 Å throughout the simulation after a stabilization period of 50 ns. There were fluctuations in the RMSD value throughout the whole MD run of 200 ns and a difference of ~ 2 Å to ~ 3 Å was observed between the backbone and complex. The RMSF value was found to be in the range of ~ 3 Å to ~ 7 Å; a high-rise peak (~ 3 Å to ~ 7 Å) was observed between atoms ~ 1000 to ~ 1300. The radius of gyration ranged between 14Å and 17Å after the stabilization of the complex at ~ 75 ns. A highest of 4 hydrogen bonds were observed during the MD run which were not constant throughout the simulation. The solvent assessable surface area ranged between ~ 70 nm^2^ to ~ 85 nm^2^; there was an observed increase in the SASA after 140 ns of MD run which may be due to unstable hydrogen bonds formed during the MD run (Fig. 8).

### Molecular mechanics Poisson-Boltzmann surface area (MMPBSA) analysis

MMPBSA analysis performed for 82 frames for all the six complexes revealed that the vander waals and electrostatic molecular mechanics energy were found to be least for β-catenin complex (− 0.11 ± 0.13 kcal/mol & − 36.49 ± 0.24 kcal/mol respectively) whereas, cytochrome 1A1 and sirtuin 1 complexes possessed the highest i.e. 0.58 ± 0.14 kcal/mol and − 35.23 ± 0.19 kcal/mol respectively. Moreover, total gas phase and total salvation energy were found to be lowest by β-catenin-sinapic acid and *PRKCA* complex i.e. 50.57 ± 0.61 and − 29.35 ± 0.11 kcal/mol respectively; whereas, the highest was found to be by *CYP3A4* and β-catenin i.e. 54.47 ± 0.36 and − 25.74 ± 0.41 kcal/mol respectively. The total binding energy was found to be least for cytochrome 1A1 (23.92 ± 0.36 kcal/mol) indicating it to be the most stable complex (Table 2).

**Table 2:** MMPBSA analysis of hub genes & sinapic acid as a complex.

Protein	∆VDWAALS	∆EEL	∆EPB	∆ENPOLAR	∆EDISPER	∆GGAS	∆GSOLV	∆GTotal
PRKCA	0.16 ± 0.13	− 34.91 ± 0.20	− 30.97 ± 0.11	26.76 ± 0.02	− 25.14 ± 0.02	53.83 ± 0.35	− 29.35 ± 0.11	24.48 ± 0.35
CASP8	0.09 ± 0.12	− 34.91 ± 0.19	− 30.80 ± 0.19	26.76 ± 0.02	− 25.18 ± 0.02	53.95 ± 0.41	− 29.23 ± 0.12	24.72 ± 0.43
SIRT1	0.35 ± 0.14	− 34.82 ± 0.18	− 30.79 ± 0.09	26.74 ± 0.02	− 25.17 ± 0.02	53.9 ± 0.46	− 29.22 ± 0.09	24.67 ± 0.46
CYP1A1	0.58 ± 0.14	− 35.23 ± 0.19	− 30.82 ± 0.10	26.72 ± 0.01	− 25.22 ± 0.02	53.23 ± 0.37	− 29.31 ± 0.10	23.92 ± 0.36
CYP3A4	− 0.09 ± 0.14	− 35.38 ± 0.23	− 30.42 ± 0.17	26.75 ± 0.02	− 25.16 ± 0.02	54.47 ± 0.36	− 28.83 ± 0.17	25.65 ± 0.36
CTNNB1	− 0.11 ± 0.13	− 36.49 ± 0.24	− 27.32 ± 0.41	26.76 ± 0.02	− 25.18 ± 0.02	50.57 ± 0.61	− 25.74 ± 0.41	24.83 ± 0.40

All the data are presented in mean ± SEM (n = 82) and unit for each parameter is kcal/mol.

∆*VDWAALS* Vander Waals molecular mechanics energy, ∆*EEL* electrostatic molecular mechanics energy, ∆*EPB* polar contribution to the salvation energy, ∆*ENPOLAR* non-polar contribution of solute–solvent interactions to the solvation energy, ∆*EDISPER* non-polar contribution of attractive solute–solvent interactions to the salvation energy, ∆*GGAS* total gas phase molecular mechanics energy, ∆*GSOLV* total solvation energy, ∆*GTotal* total relative binding energy.

## Discussion

The present study aimed to propose the possible molecular mechanism of sinapic acid in the treatment of breast cancer via integrating multiple system biology tools like gene set enrichment analysis, gene ontology, and molecular dynamic simulation. Sinapic acid has been reported to possess time and dose-dependent suppressive action on breast and colon cancer cell lines representing its potential to be a chemotherapeutic drug to be used for the treatment of breast cancer^37^. Also, it has been reported to possess an ameliorating effect on various organ toxicities if used in combination with chemotherapeutic drugs like cisplatin, doxorubicin, and methotrexate^24–26^. Sinapic acid being a plant metabolite is reported to possess ameliorating effect on various organ toxicities by chemotherapeutic agents via the regulation of *Nrf2*/*HO*-1 involved in the *NF*-*κB* signalling pathway^25^.

Initially, we acquired targets involved in breast cancer through DisGeNET database where we identified 6776 genes to be involved in the pathogenesis of breast cancer, which were further matched with proteins predicted to be modulated by sinapic acid; identified from DIGEP-Pred database. These matched proteins (62 proteins) were used to construct a protein–protein interaction network via STRING. KEGG pathway analysis was used to identify various pathways being regulated by sinapic acid; 50 pathways were identified to be involved. Further, a protein-pathway network was constructed using Cytoscape *ver. 3.9.0* from which three proteins were identified to be lead hits (*CASP8*, *CTNNB1*, and *PRKCA*). GO analysis was performed based on three GO terms CC, MF, and BP. In addition, Cluster analysis was also performed to identify various groups of proteins and how they belong to a category biologically^38^. Molecular docking was performed with three different docking tools *i.e.* AutoDock 4.2, AutoDock Vina, and Schrodinger suite glide to attain better visibility of ligand–protein docking with matched 62 targets.

Gene ontology analysis revealed “*extracellular space*”, “*protein binding*”, and “*response to chemical*” to possess the least false discovery rate in GO analysis; CC, MF, and BP respectively. Also, we predicted that 79% of genes involved in GO and KEGG mediated proteins were in common which represents a good interaction between the GO terms and KEGG mediated proteins. Further, cluster analysis revealed the presence of “*cellular response to oxidative stress*” to be majorly modulated by the most number of genes (36 genes).

We performed docking on 62 targets with sinapic acid using different software’s; it was predicted that cytochrome enzymes (*CYP1A1*, *CYP3A4*, and *CYP3A7*), *CAT*, *SIRT1*, *VDR*, and *NOS2* possessed the lowest binding energy with the ligand sinapic acid. The molecular dynamic simulation revealed that all complexes became stable after the stabilization period (~ 50 ns to ~ 100 ns); a maximum of ~ 2.5 Å to ~ 3.0 Å difference was observed in the RMSD value of the backbone and complex. The complexes of sirtuin 1 & β-catenin with sinapic acid possessed a difference in RMSD values between backbone and complex ~ 2.5 Å which falls in an acceptable range. However, there was a fluctuation in the number of hydrogen bonds although the difference in RMSD values were within the range of ~ 3 Å, this may be due to other hydrophobic and Vander Waal interactions between the ligand and proteins^39^. Similarly, we observed in the MMPBSA that the complexes possessed low Vander Waal molecular mechanics energy indicating Vander Waal forces to be acting and making the complex stable.

**Figure 9:**
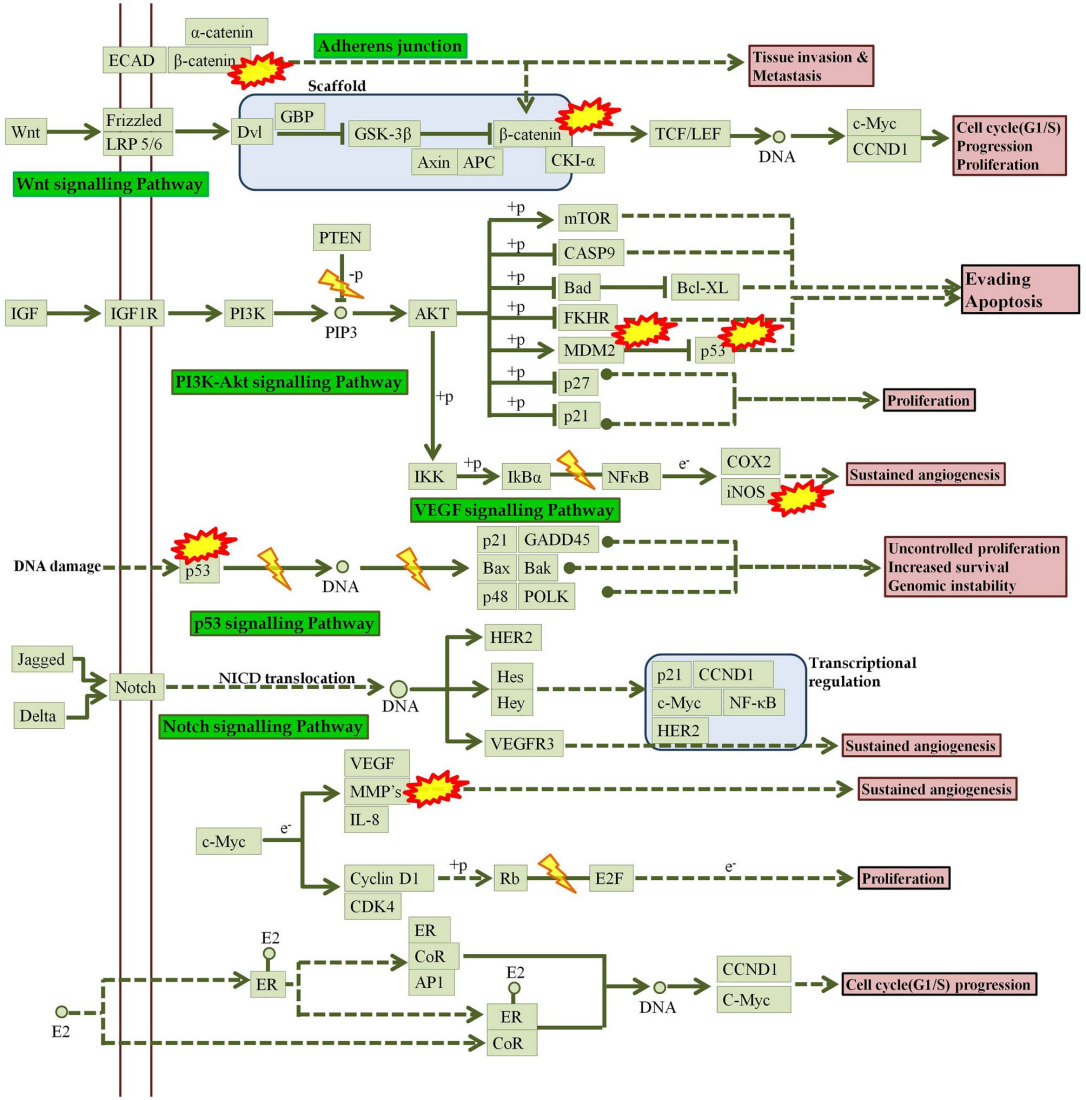
Proposed possible molecular mechanism of sinapic acid in breast cancer.  Indicates genes identified to be modulated by sinapic acid^57^.

Network pharmacology analysis revealed classical protein kinase C α-type (*PRKCA*) to be involved in majority of pathways identified in breast cancer. Protein kinase C belongs to the class of serine/threonine protein kinase family of enzymes and plays a vital role in the progression of several diseases like cancer, diabetes, autoimmune diseases, heart failure, and Parkinsonism^40^. It has been reported that the expression of *PRKCA* is associated with endocrine resistance and poor prognosis in ER-positive (ER +) breast tumors. In addition, expression of *PRKCA* is elevated in triple negative breast cancer (TNBC) patients and shown to be responsible for chemotherapy resistance and metastasis^41^. *PKCα* acts as an upstream regulator of *FOXC2*, which in turn represses the expression of p120-catenin, an important component of adherens junction that acts as the anchor for E-cadherin^42^. Hence, *PRKCA* could be a potent target to be suppressed for a better treatment strategy to control breast cancer. However, there is still scope to understand the role of *PRKCA* in breast cancer as much is still to be uncovered. Figure 9 represents the predicted probable molecular mechanism of sinapic acid for the treatment of breast cancer.

In addition, *CASP8* and *CTNNB1* managed to be in the top 3 lead hits identified via network pharmacology; *CASP8* is found to be down-regulated in breast cancer due to promoter methylation^43^. *CASP8* is an important initiator of apoptosis. Absence or down-regulation of *CASP8* could cause resistance to apoptosis and is correlated with unfavorable disease outcomes, such as childhood medulloblastoma and neuroblastoma. The absence or down-regulation of *CASP8* may be due to epigenetic changes^44^. Hence, promoting *CASP8* expression might help well in the treatment of breast cancer. Similarly, role of *CTNNB1* after birth, *WNT*/*CTNNB1* responsive stem cells are responsible for tissue homeostasis in various organs and hyperactive *WNT*/*CTNNB1* signaling is observed in many different human cancers^45^. The first link between *WNT* signaling and breast cancer was established when *WNT1* was identified as a proto-oncogene capable of driving mammary tumor formation in mice^46^. However, much debate and controversy persist regarding the importance of *WNT* signaling for the initiation, progression, or maintenance of different breast cancer subtypes^47^. The upregulation of β-catenin and its accumulation in the nucleus promotes the transcription of genes like *c-Myc* & cyclin D-1^48^. Hence, *CTNNB1* was predicted to be down-regulated which may be one of the mechanisms by which sinapic acid prevents the progression of cancer.

Cytochrome enzymes play a major role in the activation of drugs to activated carcinogens from which *CYP1A1* enzyme plays a major role as continuous exposure to inhalation chemicals and environmental carcinogens are thought to increase the level of *CYP1A1* expression in extrahepatic tissues, through the aryl hydrocarbon receptor (AhR); may be due to the tendency of *CYP1A1* to metabolize carcinogens. Reports suggest that upregulation of *CYP3A4* is seen in 80% of breast tumors and can be used to identify tumor response in different treatments^49^.

Moreover, catalase (*CAT*) is an antioxidant enzyme that catalyzes mainly the transformation of hydrogen peroxide into water and oxygen^50^. Although catalase is frequently down-regulated in tumors the underlying mechanism remains unclear. Further, silent information regulation factor 1 (sirtuin Type 1, *SIRT1*), as a kind of NAD + dependent class III histone deacetylation enzyme, involved in tumor proliferation, invasion, and metastasis^51^. The roles of *SIRT1* in breast cancer is multifaceted depending on its substrate from upstream or downstream signaling pathway. Results have displayed that *SIRT1* is significantly up-regulated in breast cancer tissues and cells, which is correlated with histological grade, tumor size, and lymph node metastasis^52^. Studies report that vitamin D has been suggested to prevent and improve the prognosis of several cancers, including breast cancer^53^; high expression of *VDR* in invasive breast tumors is associated with favorable prognostic factors and a low risk of breast cancer death^54^. Hence, a high *VDR* expression is a positive prognostic factor which may be used to identify response of tumors in different treatments. Expression of inducible nitric oxide synthase (*NOS2*) has been associated with poor outcome in breast cancer^55^; the upregulation of *NOS* leads to tumor angiogenesis by upregulating *VEGF*^56^.

## Conclusion

The present study aimed to identify a possible molecular mechanism of sinapic acid (SA) in the treatment of breast cancer via the utilization of multiple system biology tools like network pharmacology, Gene ontology enrichment, and molecular dynamic simulation. The predictions revealed that the mechanism of sinapic acid in breast cancer may be due to multiple pathways and proteins like β-catenin, *PRKCA*, *CASP8,* and cytochrome enzymes (*CYP1A1* and *CYP3A4*); the majorly regulated pathway was predicted to be “*Pathways in cancer*”. This indicates that sinapic acid can be used in the treatment of breast cancer. However, these are predictions based on previous reports, which need to be validated and looked upon in-depth to confirm the exact molecular mechanism of sinapic acid in the treatment of breast cancer; this is future scope as well as a drawback of the current study.

### Supplementary Information

Supplementary Information.

## Data Availability

The datasets generated and/or analysed during the current study are available in the UniProt repository with accession links: https://www.uniprot.org/uniprotkb/O95352/entry, https://www.uniprot.org/uniprotkb/P35222/entry, https://www.uniprot.org/uniprotkb/Q9GZT9/entry, https://www.uniprot.org/uniprotkb/P02724/entry, https://www.uniprot.org/uniprotkb/P34932/entry, https://www.uniprot.org/uniprotkb/P41134/entry, https://www.uniprot.org/uniprotkb/P05783/entry, https://www.uniprot.org/uniprotkb/P08727/entry, https://www.uniprot.org/uniprotkb/P08729/entry, https://www.uniprot.org/uniprotkb/P05787/entry, https://www.uniprot.org/uniprotkb/P08138/entry, https://www.uniprot.org/uniprotkb/P16860/entry, https://www.uniprot.org/uniprotkb/Q9NRD5/entry, https://www.uniprot.org/uniprotkb/Q9Y3F4/entry, https://www.uniprot.org/uniprotkb/P20366/entry, https://www.uniprot.org/uniprotkb/P01033/entry, and https://www.uniprot.org/uniprotkb/O15350/entry. The targets predicted to be modulated by sinapic acid were retrieved from the DIGEP-Pred database (http://www.way2drug.com/ge/). Additionally, the targets involved in the pathogenesis of breast cancer were retrieved from the DisGeNET database (https://www.disgenet.org/) with disease id C0678222.

## Abbreviations

AhR: Aryl hydrocarbon receptor
BC: Breast cancer
BP: Biological process
CC: Cellular component
GO: Gene ontology
ER: Estrogen receptor
FDR: False discovery rate
GC: Gene count
HRT: Hormone replacement therapy
KEGG: Kyoto encyclopedia of genes and genomes
MD: Molecular dynamics
MF: Molecular function
MMPBSA: Molecular mechanics Poisson-boltzmann surface area
NAD: Nicotinamide adenine dinucleotide
NBHA: Number of hydrogen bond acceptor
NHBD: Number of hydrogen bond donor
NOS: Nitric oxide synthase
PDB: Protein data bank
Rg: Radius of gyration
RMSD: Root mean square deviation
RMSF: Roost mean square fluctuation
RCSB: Research collaboratory for structural bioinformatics
SA: Sinapic acid
SMILES: Simplified molecular input line entry system
TNBC: Triple negative breast cancer
XP: Extra precision

## References

[1] Wong RS (2011). Apoptosis in cancer: From pathogenesis to treatment. J. Exp. Clin. Cancer Res..

[2] Sharma GN (2010). Various types and management of breast cancer: An overview. J. Adv. Pharm. Technol. Res..

[3] Sung H (2021). Global cancer statistics 2020: GLOBOCAN estimates of incidence and mortality worldwide for 36 cancers in 185 countries. CA Cancer J. Clin..

[4] Rahimzadeh S (2021). Geographical and socioeconomic inequalities in female breast cancer incidence and mortality in Iran: A Bayesian spatial analysis of registry data. PLoS One.

[5] Feng Y (2018). Breast cancer development and progression: Risk factors, cancer stem cells, signaling pathways, genomics, and molecular pathogenesis. Genes Dis..

[6] McVeigh UM, Tepper JW, McVeigh TP (2021). A review of breast cancer risk factors in adolescents and young adults. Cancers.

[7] Dwivedi PSR (2022). System biology-based investigation of Silymarin to trace hepatoprotective effect. Comput. Biol. Med..

[8] Dwivedi PSR (2021). Gene set enrichment analysis of PPAR-γ regulators from *Murraya*
*odorata* Blanco. J. Diabetes Metab. Disord..

[9] Dwivedi PSR (2021). Exploring the therapeutic mechanisms of Cassia glauca in diabetes mellitus through network pharmacology, molecular docking and molecular dynamics. RSC Adv..

[10] Patil A (2022). GLUT-2 mediated glucose uptake analysis of Duranta repens: In-silico and In-vitro approach. J. Diabetes Metab. Disord..

[11] Sharifi-Rad J (2021). Paclitaxel: Application in modern oncology and nanomedicine-based cancer therapy. Oxid. Med. Cell Longev..

[12] Arora RA (2010). Anticancer alkaloids of *Catharanthus*
*roseus*: Transition from traditional to modern medicine. Herb. Med. Cancer Chemoprev. Ther. Perspect..

[13] Nićiforović N, Abramovič H (2014). Sinapic acid and its derivatives: Natural sources and bioactivity. Compr. Rev. Food Sci. Food Saf..

[14] Razzaghi-Asl N (2013). Antioxidant properties of hydroxycinnamic acids: A review of structure-activity relationships. Curr. Med. Chem..

[15] Balaji C (2014). Chemopreventive effect of sinapic acid on 1,2-dimethylhydrazine-induced experimental rat colon carcinogenesis. Hum. Exp. Toxicol..

[16] Eroğlu C (2018). Anticancer mechanism of sinapic acid in PC-3 and LNCaP human prostate cancer cell lines. Gene.

[17] Janakiraman K (2015). Influence of sinapic acid on induction of apoptosis in human laryngeal carcinoma cell line. Int. J. Mod. Res. Rev..

[18] Hameed H (2016). Assessment of cytotoxic properties of sinapic acid in vitro. Turk. J. Pharm. Sci..

[19] RajPreeth D (2019). Green synthesis of copper oxide nanoparticles using sinapic acid: An underpinning step towards antiangiogenic therapy for breast cancer. J. Biol. Inorg. Chem..

[20] Kampa M (2004). Antiproliferative and apoptotic effects of selective phenolic acids on T47D human breast cancer cells: Potential mechanisms of action. Breast Cancer Res..

[21] Ternikar SG (2021). Gene ontology enrichment analysis of PPAR-γ modulators from *Cassia*
*glauca* in diabetes mellitus. J. Diabetes Metab. Disord..

[22] Dwivedi PSR (2021). Identification of PTP1B regulators from *Cymbopogon*
*citratus* and its enrichment analysis for diabetes mellitus. In Silico Pharmacol..

[23] Virk JK (2020). Isolation of sinapic acid from Habenaria intermedia D. Don: A new chemical marker for the identification of adulteration and substitution. Curr. Tradit. Med..

[24] Bin-Jardan YA (2020). Sinapic acid ameliorates oxidative stress, inflammation, and apoptosis in acute doxorubicin-induced cardiotoxicity *via* the NF-κB-mediated pathway. Biomed. Res. Int..

[25] Ansari MA (2017). Sinapic acid modulates Nrf2/HO-1 signaling pathway in cisplatin-induced nephrotoxicity in rats. Biomed. Pharmacother..

[26] Ahmad A (2021). Sinapic acid mitigates methotrexate-induced hepatic injuries in rats through modulation of Nrf-2/HO-1 signaling. Environ. Toxicol..

[27] Lagunin A (2013). DIGEP-Pred: Web service for in silico prediction of drug-induced gene expression profiles based on structural formula. Bioinformatics.

[28] Szklarczyk D (2016). The STRING database in 2017: Quality-controlled protein–protein association networks, made broadly accessible. Nucleic Acids Res..

[29] Shannon P (2003). Cytoscape: A software environment for integrated models of biomolecular interaction networks. Genome Res..

[30] Bindea G (2009). ClueGO: A Cytoscape plug-in to decipher functionally grouped gene ontology and pathway annotation networks. Bioinformatics.

[31] Halgren TA (1996). Merck molecular force field. I. Basis, form, scope, parameterization, and performance of MMFF94. J. Comput. Chem..

[32] Guex N, Peitsch MC (1997). SWISS-MODEL and the Swiss-Pdb Viewer: An environment for comparative protein modeling. Electrophoresis.

[33] Vanommeslaeghe K (2010). CHARMM general force field: A force field for drug-like molecules compatible with the CHARMM all-atom additive biological force fields. J. Comput. Chem..

[34] Dwivedi PSR, Shastry CS (2023). Anti-tumor potential and mode of action of Karanjin against breast cancer; an in-silico approach. Arab. J. Chem..

[35] Valdés-Tresanco MS (2021). gmx_MMPBSA: A new tool to perform end-state free energy calculations with GROMACS. J. Chem. Theory Comput..

[36] Kumari R (2014). g_mmpbsa- A GROMACS tool for high-throughput MM-PBSA calculations. J. Chem. Inf. Model..

[37] Kampa M (2004). Antiproliferative and apoptotic effects of selective phenolic acids on T47D human breast cancer cells: Potential mechanisms of action. Breast Cancer Res..

[38] Hu CW (2015). Progeny clustering: A method to identify biological phenotypes. Sci. Rep..

[39] Khanal P (2022). Computational investigation of benzalacetophenone derivatives against SARS-CoV-2 as potential multi-target bioactive compounds. Comput. Biol. Med..

[40] Mochly-Rosen D, Das K, Grimes KV (2012). Protein kinase C, an elusive therapeutic target?. Nat. Rev. Drug Discov..

[41] Tonetti DA (2012). PKC and ER are associated with triple-negative breast cancers in African American and Caucasian patients. Int. J. Breast Cancer.

[42] Pham TND (2017). Protein kinase C α enhances migration of breast cancer cells through FOXC2-mediated repression of p120-catenin. BMC Cancer.

[43] Wu Y (2010). Caspase 8 and maspin are downregulated in breast cancer cells due to CpG site promoter methylation. BMC Cancer.

[44] Pistritto G (2016). Apoptosis as anticancer mechanism: Function and dysfunction of its modulators and targeted therapeutic strategies. Aging (Albany NY).

[45] van Schie EH, van Amerongen R (2020). Aberrant WNT/CTNNB1 signaling as a therapeutic target in human breast cancer: Weighing the evidence. Front. Cell Dev. Biol..

[46] Brown AM (2001). Wnt signaling in breast cancer: Have we come full circle?. Breast Cancer Res..

[47] Xu X (2020). Wnt signaling in breast cancer: Biological mechanisms, challenges and opportunities. Mol. Cancer.

[48] Shang S, Hua F, Hu ZW (2017). The regulation of β-catenin activity and function in cancer: Therapeutic opportunities. Oncotarget.

[49] Androutsopoulos VP, Tsatsakis AM, Spandidos DA (2009). Cytochrome P450 CYP1A1: Wider roles in cancer progression and prevention. BMC Cancer.

[50] Nandi A (2019). Role of catalase in oxidative stress- and age-associated degenerative diseases. Oxid. Med. Cell Longev..

[51] Heck DE (2010). Mechanisms of oxidant generation by catalase. Ann. N. Y. Acad. Sci..

[52] Jin X (2018). SIRT1 promotes formation of breast cancer through modulating Akt activity. J. Cancer.

[53] Feldman D (2014). The role of vitamin D in reducing cancer risk and progression. Nat. Rev. Cancer.

[54] Huss L (2019). Vitamin D receptor expression in invasive breast tumors and breast cancer survival. Breast Cancer Res..

[55] Ridnour LA (2012). Nitric oxide synthase and breast cancer: Role of TIMP-1 in NO-mediated Akt activation. PLoS One.

[56] Hood JD (1998). VEGF upregulates ecNOS message, protein, and NO production in human endothelial cells. Am. J. Physiol..

[57] Kanehisa M, Goto S (2000). KEGG: Kyoto encyclopedia of genes and genomes. Nucleic Acids Res..

