# Supplementary material for: System biology mediated assessment of molecular mechanism for sinapic acid against breast cancer: via network pharmacology and molecular dynamic simulation
Source: Sci Rep. 2023 Dec 11;13:21982. doi: 10.1038/s41598-023-47901-3 (PMC10713517; doi:10.1038/s41598-023-47901-3)
Supplement: Supplementary file 1 — Supplementary Information. [file 41598_2023_47901_MOESM1_ESM.zip › Supplimentary files/Supplementary File Footers.docx]

**Supplementary File Footer:**

**Supplementary File 1:** Gene enrichment and Network analysis of sinapic acid against breast cancer

**Supplementary File 2:** Gene Ontology analysis of sinapic acid (Cellular component (Sheet 1), Molecular function (Sheet 2), and Biological process (Sheet 3)) against breast cancer

**Supplementary file 3:** Cluster analysis of KEGG enriched genes and gene ontology of Sinapic acid against breast cancer

**Supplementary file 4:** Target properties and Molecular docking analysis of sinapic acid with hub genes *via* AutoDock Vina, AutoDock 4.2, and Schrodinger suite Glide Availability of data

**Supplementary file 5:** The cluster analysis of KEGG identified gene ontology and pathways for sinapic acid against breast cancer
